# Perceived Duration: The Interplay of Top-Down Attention and Task-Relevant Information

**DOI:** 10.3389/fpsyg.2019.00490

**Published:** 2019-03-06

**Authors:** Alejandra Ciria, Florente López, Bruno Lara

**Affiliations:** ^1^Facultad de Psicología, Universidad Nacional Autónoma de México, Mexico City, Mexico; ^2^Laboratorio de Robótica Cognitiva, Centro de Investigación en Ciencias, Universidad Autónoma del Estado de Morelos, Cuernavaca, Mexico

**Keywords:** perceived duration, oddball effect, top-down attention, task-relevant information, stimulus expectations, saliency

## Abstract

Perception of time is susceptible to distortions; among other factors, it has been suggested that the perceived duration of a stimulus is affected by the observer’s expectations. It has been hypothesized that the duration of an oddball stimulus is overestimated because it is unexpected, whereas repeated stimuli have a shorter perceived duration because they are expected. However, recent findings suggest instead that fulfilled expectations about a stimulus elicit an increase in perceived duration, and that the oddball effect occurs because the oddball is a target stimulus, not because it is unexpected. Therefore, it has been suggested that top-down attention is sometimes sufficient to explain this effect, and sometimes only necessary, with an additional contribution from saliency. However, how the expectedness of a target stimulus and its salient features affect its perceived duration is still an open question. In the present study, participants’ expectations about and the saliency of target stimuli were orthogonally manipulated with stimuli presented on a short (Experiment 1) or long (Experiment 2) temporal scale. Four repetitive standard stimuli preceded each target stimulus in a task in which participants judged whether the target was longer or shorter in duration than the standards. Engagement of top-down attention to target stimuli increased their perceived duration to the same extent irrespective of their expectedness. A small but significant additional contribution to this effect from the saliency of target stimuli was dependent on the temporal scale of stimulus presentation. In Experiment 1, saliency only significantly increased perceived duration in the case of expected target stimuli. In contrast, in Experiment 2, saliency exerted a significant effect on the overestimation elicited by unexpected target stimuli, but the contribution of this variable was eliminated in the case of expected target stimuli. These findings point to top-down attention as the primary cognitive mechanism underlying the perceptual extraction and processing of task-relevant information, which may be strongly correlated with perceived duration. Furthermore, the scalar properties of timing were observed, favoring the pacemaker-accumulator model of timing as the underlying timing mechanism.

## Introduction

*Time perception* refers to our subjective experience of both the passage of time and how much time has passed, such as the perceived duration of events (Buonomano, 2017). Although time perception is crucial for behavior, it is prone to subjective distortions. Modifications to the physical properties of stimuli, among other factors, can induce distortions in the perception of objective time (for a review, see Eagleman, 2008). The fact that perceived duration is affected by the observer’s expectations about stimuli is of particular interest here; how this phenomenon operates is still an open question. There is existing evidence to suggest that fulfilled expectations reduce (Tse et al., 2004; Ulrich et al., 2006; Pariyadath and Eagleman, 2007, 2012; Matthews, 2011; Schindel et al., 2011), do not reduce (van Wassenhove et al., 2008; Cai et al., 2015), or even increase perceived duration of a stimulus (Matthews’, 2015; Matthews and Gheorghiu, 2016; Matthews and Meck, 2016; Schweitzer et al., 2017; Skylark and Gheorghiu, 2017; Birngruber et al., 2018).

The effects of expectations on perceived duration have been found to be closely related to repetition effects: the first stimulus in a sequence of repetitive stimuli is perceived as lasting longer than subsequent stimuli of the same physical duration (Rose and Summers, 1995; Pariyadath and Eagleman, 2007). Moreover, in a classical temporal oddball task, the duration of an unexpected deviant target stimulus (oddball) is overestimated if it is presented randomly within a train of repetitive stimuli with a constant standard duration (Tse et al., 2004; Pariyadath and Eagleman, 2007, 2012; Schindel et al., 2011; Birngruber et al., 2014). This phenomenon has been termed “time’s subjective expansion” (Tse et al., 2004), “oddball chronostasis” (Lin and Shimojo, 2017), or more usually the “temporal oddball effect” (OE; e.g., Pariyadath and Eagleman, 2007, 2012; Schindel et al., 2011; Birngruber et al., 2014, 2018; Matthews and Gheorghiu, 2016). The OE is a robust perceptual phenomenon that persists regardless of the type of temporal task used (e.g., Tse et al., 2004; Matthews, 2011; Birngruber et al., 2014). Moreover, the OE can also be elicited using variations on the classical temporal oddball task, such as presenting the target stimulus in a fixed position in the sequence of standards (e.g., van Wassenhove et al., 2008; Herai and Mogi, 2010; Cai et al., 2015), or even using a comparison task, in which only one standard stimulus precedes the target stimuli (e.g., Ulrich et al., 2006; Matthews, 2011; Matthews’, 2015; Skylark and Gheorghiu, 2017).

Three main explanatory accounts have been proposed of the emergence of the OE; these can be grouped into two major theories based on their core explanatory assumptions. First, attentional and arousal accounts of the OE have been suggested based on the pacemaker-accumulator model, which assumes the existence of a specialized internal clock mechanism for timing processes. The pacemaker-accumulator model is the dominant paradigm in the timing literature (Gibbon and Church, 1984; Gibbon, 1991; Church, 2003; Buhusi and Meck, 2005), mainly because timing ability shares many characteristic hallmarks with sensory perception, such as scalar properties (Wearden and Lejeune, 2008; Allman et al., 2014). The second major explanatory theory is the predictive coding account, centered on the neural coding efficiency framework. The core explanatory hypothesis is that perceived duration correlates with the size of the evoked neural response to a stimulus (Pariyadath and Eagleman, 2007, 2012; Eagleman and Pariyadath, 2009; Matthews, 2011; Schindel et al., 2011; Matthews et al., 2014).

The attentional account of the OE proposes that the engagement of attention by an oddball stimulus triggers an increase in perceptual information processing, which accelerates the internal pacemaker, and as a consequence increases the perceived duration of the stimulus (Tse et al., 2004). From a mechanistic standpoint, the internal clock has a switch and a pacemaker-accumulator mechanism that emits and accumulates pulses determining perceived duration. With the onset of a stimulus to be timed, the switch closes, establishing a connection between the pacemaker mechanism and the accumulator in order to store the pulses emitted by the pacemaker (Treisman, 1963; Gibbon, 1977; Church, 1984; Gibbon and Church, 1984). In a series of experiments, Tse et al. (2004) explored the OE using different types of oddball stimuli (e.g., expanding black disks, black stationary disks, colored disks, squares) presented within a series of repetitive standards. They report that the saliency (defined operationally as the amount of change) of an oddball stimulus modulates the magnitude of the OE. Under this view, the internal pacemaker has access to the rate of perceptual information processing, so when more attention is drawn toward an oddball stimulus, more pulses are stored. The authors suggest that the magnitude of the OE should increase with the oddness or improbability of a stimulus, and with the presence of more salient physical properties.

A similar theoretical account, also based on the pacemaker-accumulator model, proposes that an unexpected stimulus increases the observer’s level of arousal, resulting in the accumulation of more pulses and thus in an overestimation of the duration of the oddball (Ulrich et al., 2006; New and Scholl, 2009). For example, Ulrich et al. (2006) used a comparison task to manipulate expectations by presenting oddball stimuli with high and low frequency. Their results support the idea that infrequent stimuli raise the observer’s level of arousal and thus the apparent duration of such stimuli. The OE occurs because unexpected stimuli are especially arousing, increasing the rate of information processing, which in turn accelerates the internal pacemaker. These effects may arise because attention drives arousal, in turn accelerating the pulse rate (Treisman, 1963).

Instead of postulating a specialized internal clock mechanism to explain timing processes, the predictive coding account of the OE suggests that perceived duration correlates with the evoked neural response that reflects how efficiently a stimulus has been coded. Under this framework, stimulus repetition or, more generally, fulfilled expectations about stimulus features, contracts perceived duration as a result of a reduction in the evoked neural response, so that an oddball or unexpected stimulus appears longer in duration in comparison with repeated stimuli (Pariyadath and Eagleman, 2007, 2012). The neural suppression induced by an expected stimulus occurs as a consequence of the acquisition of a more efficient representation of the stimulus (Pariyadath and Eagleman, 2012), decreasing metabolic costs (Grill-Spector et al., 2006). In fact, the number of repetitions of the standard stimulus modulates the magnitude of the OE (Pariyadath and Eagleman, 2012), and the latter increases monotonically with the degree of discrepancy between the standard stimuli and the oddball (Schindel et al., 2011; Pariyadath and Eagleman, 2012). Accordingly, the perceived duration of a stimulus may reflect the magnitude of the discrepancy between expected and actual sensorial input (prediction error), or how efficiently that stimulus was coded (Pariyadath and Eagleman, 2007, 2012; Schindel et al., 2011). Under this framework, the OE is not fundamentally an attention-related process, but rather a predictive process in which the violation of prediction makes an oddball odd and, as a consequence, exogenous attention is drawn toward it (Pariyadath and Eagleman, 2007, 2012; Schindel et al., 2011).

Although they explain the effect differently, the attentional, arousal, and predictive coding accounts share the assumption that an oddball stimulus is perceived as lasting longer because it is unexpected and repeated stimuli are perceived as shorter in duration because they are expected. These expectation-based accounts agree that the rate of information processing is tightly associated with the perceived duration of a stimulus. In this line of thought, these theories suggest that the predictability of a stimulus is crucial in determining the direction of its perceived duration relative to physical duration. Therefore, an unexpected stimulus has an increased perceived duration because more processing resources are required to extract the perceptual information from the stimulus. In contrast, an expected stimulus has a contracted perceived duration because the fact that features of the stimulus are already anticipated reduces the amount of perceptual information that has to be extracted. However, recent empirical evidence, based on studies in which different forms of expectations are experimentally manipulated, has challenged these assumptions about the effects of expectations on perceived duration.

Cai et al. (2015) experimentally manipulated different types of expectations by presenting one type of sequence more frequently than another, by changing the regularity of stimuli in a sequence, and by presenting stimuli from overlearned sequences. They consistently found that perceived duration is only reduced with repetition of stimuli, and not in accordance with participants’ expectations of the sequence configuration. Nevertheless, they observed that the repetition effect was diminished when repeats were more frequent (80% compared to 20%). Furthermore, Matthews’ (2015) manipulated expectations by varying the frequency of repetitions within blocks of trials using a comparison task, presenting only two consecutive stimuli, of which the second was either repeated or different. The design consisted of high repetition rate blocks (80% repeated vs. 20% different) and low repetition rate blocks. Mirroring Cai et al.’s (2015) findings, when the probability of repetition of the target stimulus was higher, the repetition effect was eliminated or even reversed. Matthews’ (2015) suggests that repetition of stimuli exerts opposing effects on perceived duration: a first-order repetition effect, related to a low-level adaptation process, reduces perceived duration, while a higher-order repetition effect, which is associated with expectations about repetition, increases perceived duration. Skylark and Gheorghiu (2017) replicated Matthews’ (2015) study, reporting the same pattern of results using slightly different stimuli and different durations for the first and second stimuli.

To isolate the effects of stimulus expectations on perceived duration from those of mere repetition and probability, Birngruber et al. (2018) used the self-generated expectations paradigm (Bernstein and Reese, 1965). During each trial, participants vocalized their expectations about the color or shape of the target stimulus and then performed a temporal bisection task, categorizing the stimuli as “rather short” or “rather long.” They found that when a target stimulus matched the participant’s self-generated expectation, it was judged to last longer than when it did not. In addition to self-generated expectations, it is essential to consider that expectations are frequently built via associative processes between objects and the context in which they appear (Bar, 2007). Schweitzer et al. (2017) used a classical temporal oddball task to replicate the OE, presenting either contextually associated or non-associated oddballs. However, the duration of the oddball was overestimated to a greater extent in the case of contextually associated information. The authors argue that top-down attention is enhanced by an effect in which contextually associated oddballs increase the rate of perceptual information processing.

Together, these recent experiments have led to the conclusion that expectations do not reduce perceived duration. In fact, contrary to the claims of expectation-based accounts, these studies suggest that fulfilled expectations about a stimulus increase its perceived duration. However, direct comparison between findings is a difficult task, given the notable methodological differences among the experiments described above. Furthermore, some of these experimental methods interfere with explanatory assumptions about how expectations affect perceived duration. For example, Cai et al. (2015) randomly alternated different target stimuli within blocks, presenting interleaved trials with different types of target stimulus, meaning that participants’ expectations about the target stimulus could have been inadvertently affected. In an experiment presenting overlearned sequences of numbers (e.g., 1-2-3-4) as standard stimuli, participants had the opportunity to learn that the number following 4 would not always be a 5, because one of the numbers 4, 5, or 6 was randomly presented as target stimulus on each such trial. Therefore, the occurrence of a particular previously expected number (in this case, 5) as the target stimulus on each trial became unexpected. Moreover, the majority of the above-mentioned experiments (e.g., Matthews’, 2015; Skylark and Gheorghiu, 2017) used a comparison task, so it is unclear whether the same pattern of results would also occur in a classic temporal oddball task. Although Schweitzer et al. (2017) used a classic temporal oddball task, they randomly presented the contextually associated and non-associated oddballs within a block, and in each trial the oddball could appear at any position in the sequence except first or last. In this case, regardless of the top-down attentional effects of contextual associations that the authors observed, it can be argued that both contextually associated and non-associated oddballs were unexpected. Additionally, if expectations increase perceived duration, it is not clear why in a classic temporal oddball task a target stimulus is perceived as lasting longer when compared with the preceding repeated and expected standard stimuli. In fact, higher-level expectations for standard stimuli might even diminish overestimation of the oddball’s duration (Birngruber et al., 2018). Therefore, it is still unclear how expected and unexpected target stimuli affect perceived duration.

To find an explanation for the OE, Lin and Shimojo (2017) conducted several systematic experiments to dissociate the relative contributions of each of the core explanatory factors related to the primary accounts. They suggest that the attentional account can be broken down into effects of bottom-up saliency and top-down attention, and that the predictive coding account, related to the repetition suppression effect, can be broken down into effects of adaptation processes and prediction error. Lin and Shimojo (2017) orthogonally manipulated different sequences of digits and line orientations (repeated, ordered, or random), sequences of isoluminant colors (repeated and random), and target serial positions. The configuration of stimuli in the repeated sequence was as in the classic temporal oddball task, in which all the standards are repeated and the target stimulus is distinct from the standards. In this case of repeated sequences, all three factors (top-down attention, saliency/adaptation, and prediction error) could explain the emergence of the OE. However, based on the ordered sequence case, in which the standards were never repeated but followed an ordered configuration, the adaptation/saliency factor was excluded as an explanation; finally, the case involving random sequences of standard stimuli excluded the prediction error explanation, leaving the top-down attention explanation as the only possibility for the underlying cause of the effect. Regarding the manipulation of the target stimulus position, in each trial for all sequence types, the target was presented between the fourth and the sixth position in the sequence of standard stimuli. It has been reported that when a target stimulus is presented in a later position in a sequence, it is perceived to be longer in duration than a target presented in an earlier position (Pariyadath and Eagleman, 2012; Kim and McAuley, 2013). If the repetition suppression hypothesis were correct (Pariyadath and Eagleman, 2007, 2012; Schindel et al., 2011), the magnitude of the overestimation of the duration of a target stimulus arising from its position in the sequence would have been modulated only in the case of repeated sequences, and not in the case of trials involving ordered or random sequence types. Conversely, if the OE had been found to increase with target position regardless of the sequence type for standard stimuli, this would have supported the temporal preparation hypothesis (Kim and McAuley, 2013).

In their experiments using sequences of digits and line orientations, Lin and Shimojo (2017) found that the OE differed significantly between the repeated and the ordered conditions, and between the repeated and the random conditions, but not between the ordered and the random conditions. These results suggest that both top-down attention and saliency/adaptation, but not prediction error, are necessary and equal contributing factors underlying the OE. However, in the isoluminant colors conditions, there were no significant differences between repeated and random sequences. Together, these findings suggest that top-down attention is sometimes sufficient (in the case of isoluminant colors) and sometimes necessary (in the case of digits and orientations) to explain the OE. Therefore, the additional contribution from saliency/adaptation depends on the stimulus dimension. In other words, the common underlying factor in the emergence of the OE is top-down attention. Hence, the OE occurs at least partly because the oddball is a target stimulus, which has nothing to do with being odd or unexpected. Additionally, no matter the sequence type, the main effect of serial position was significant when comparing the fourth and the sixth position, but not when comparing the fifth and the sixth position. Thus, temporal preparation is a separate modulatory effect of the OE.

The above findings suggest that in a classic temporal oddball task, top-down attention toward target stimuli, along with the additional contribution of the saliency/adaptation factor, elicits the OE. Moreover, Lin and Shimojo (2017) highlight the fact that top-down attention may operate independently of predictive coding in perceived duration, because in the random sequence condition, in which the observers were pre-instructed as to which stimulus would be the target, they found that a 100% certainty target stimulus still elicited a substantial OE. However, Lin and Shimojo’s (2017) experimental design makes it difficult to dissociate bottom-up stimulus-driven attentional effects and top-down attentional effects on modulating the magnitude of the OE. In the repeated and ordered sequences conditions in each trial, the target stimulus was chosen randomly so that its physical characteristics were unexpected. Therefore, participants had to first detect the target by identifying the item that did not follow the regularity of the sequence, which allowed a top-down deployment of attention toward the task-relevant stimulus. On the other hand, in the random sequence condition, the process of identifying the target involved top-down expectations about its physical characteristics. Additionally, all target stimuli were unexpected in their position in the sequence ‘*where*,’ in their onset ‘*when*,’ (random inter stimulus interval – ISI), and in the repeated and ordered sequences conditions, in their physical characteristics. It should be potentially useful to control top-down attention by making target stimuli in ‘*where*’ and in ‘*when*’ expected in the sequence of standards to determine how their expected and unexpected physical characteristics affect perceived duration.

Regarding target stimulus salience, how more or less salient physical characteristics modulate the OE when they are expected or unexpected is not definite. It has been reported that when targets are expected and more or less salient in their physical characteristics, stimulus salience is a determining factor for subjective distortions of time perception (van Wassenhove et al., 2008). On the other hand, when targets are unexpected and more or less salient, perceived duration is overestimated to the same extent, irrespective of their salient features (Pariyadath and Eagleman, 2007; Schindel et al., 2011). It has been suggested that stimulus properties on the efficiency of non-temporal information extraction could be an utterly useful predictor of subjective duration (Matthews and Meck, 2016). In fact, top-down attentional mechanisms enable shape-specific anticipations of the expected features of a target stimulus facilitating its subsequent processing (Ansorge et al., 2010). Hence, salient expected target stimuli may require less processing time to further increase the OE in comparison with salient unexpected target stimuli. In this regard, few studies regarding the OE have examined different temporal scales of stimuli presentation under the same experimental conditions (e.g., Tse et al., 2004; Ulrich et al., 2006). However, testing these contradictory findings about expected and unexpected salient target stimulus under different temporal scales could be useful. Additionally, the scalar properties of timing should provide evidence on whether temporal discrimination sensitivity remains constant even when the rate of information processing increases (Tse et al., 2004).

The current work addressed several empirical questions. First, to what extent does the OE elicited by expected or unexpected target stimuli differ? If the common mechanism underlying the OE is top-down attention, the effect should occur irrespective of the expected or unexpected features of the target stimulus. Moreover, do salient features of a target stimulus affect the OE differently when they are expected and when they are unexpected? If saliency is an additional contributing factor in the OE, the salience of features of the target stimuli should modulate the magnitude of the OE. However, salient and unexpected target stimuli could impose a time-course processing disadvantage, taking longer to exert their influence on perceived duration relative to the amount of perceptual information to be extracted. If perceived duration correlates with the amount of perceptual information processed, to what extent is the overestimation of the duration of the target stimulus influenced by expectations and saliency dependent on the temporal scale of stimuli presentation?

Considering that top-down attention is inherent in the processing of all target stimuli, in the present experiments, instead of the manipulation of sequences of standard stimuli, participants’ expectations about and the saliency of target stimuli were manipulated. This manipulation was designed to dissociate the relative contributions of expectations and saliency of task-relevant stimuli in eliciting the OE. As a result of Lin and Shimojo’s (2017) findings, the repeated sequence type was used in all experimental conditions: that is, all the standard stimuli were repeated, but depending on the experimental condition, target stimulus features were either expected or unexpected, and were more or less salient in comparison to the standard stimuli features. The participants’ task was to judge the target stimulus duration as “longer” or “shorter” than the constant duration of the standard stimuli. In Experiment 1, we orthogonally manipulated the expectedness and saliency of target stimuli using a short temporal scale of stimulus presentation (500 ms). In Experiment 2, we replicated Experiment 1 with a long temporal scale of stimulus presentation (1,000 ms).

## Experiment 1

### Materials and Methods

Based on the assumption that top-down attention is inherent to the processing of target stimuli, it was hypothesized in Experiment 1 that both expected and unexpected target stimuli would elicit the OE. To facilitate top-down attention, participants’ temporal preparation for the onset of target stimuli was controlled by always presenting the target stimulus in the fifth position in the sequence, and by maintaining a constant ISI. Temporal expectations for the onset of a stimulus guide attention, and such expectations are directly linked to the efficiency of information processing at the perceptual level, improving stimulus detectability and discriminability (Vangkilde et al., 2012; Kim and McAuley, 2013; Matthews and Meck, 2016). Since all target stimuli were temporally expected, participants’ expectations and stimulus saliency were only manipulated in regard to the physical features of target stimuli. Hence, this experimental design allowed the dissociation of the effects of ‘*which*’ target stimuli was presented concerning information-processing and extraction of non-temporal information from expected and unexpected, and more or less salient target stimuli.

In the expected target stimulus conditions, repeated target stimuli and non-repeated but predictable target stimuli were presented. In the sequence of repetitive standard stimuli, the distinctiveness of a non-repeated but expected target stimulus should make it more salient than an expected repeated target that is identical in its physical features to the standard. This manipulation was performed to clarify the effects of saliency on perceived duration regarding expectation of repetition and general expectations of target stimuli. The hypothesis here was that expected, non-repeated target stimuli would induce a larger increase in their perceived duration than would repeated target stimuli. In the unexpected target stimuli conditions, familiar and novel stimuli were used. Unlike familiar stimuli, novel stimuli are particularly salient, and are by definition unpredictable and difficult to categorize (Courchesne et al., 1975; Schomaker, 2015). Thus, it was hypothesized that novel stimuli would induce a larger increase in their perceived duration than would familiar but unexpected stimuli. This was performed to distinguish the effects of unexpected salient features on perceived duration in regard to the amount of perceptual information processed for familiar or novel target stimuli. Unexpected target stimuli were not surprising in terms of the probability that an unexpected event would occur: their unexpectedness was constrained to their physical features.

#### Participants

Thirty members of the Biotechnology Institute of the National Autonomous University of Mexico participated in the experiment. Of them, seven were excluded from the sample because of misunderstanding the task (*n* = 5) or flat psychometric functions (*n* = 2). The final sample size was 23 participants (seven women, aged 30–66 years, *M* = 47.4 years). All participants reported being right-handed and had normal or corrected-to-normal visual acuity. Each experimental session lasted approximately 15 min, and participants completed one session on each of four consecutive days. All participants voluntarily agreed to take part in the experiment, and none received compensation in the form of payment or course credit. They were informed that the experiment was about time perception, but remained naive about the experimental hypotheses. All participants provided informed consent before participating. The experiment was approved by the Bioethics Committee of the Biotechnology Institute at the National Autonomous University of Mexico. This study was conducted in accordance with the ethical principles of the Declaration of Helsinki.

#### Apparatus and Stimuli

The experiment was programmed in PsychoPy2 1.83.01 (Peirce, 2007). The timing of the stimuli was strictly controlled, taking into consideration all precautions suggested by Garaizar et al. (2014). A laptop was used for the presentation of stimuli and recorded all responses by the participants. The LCD screen had a resolution of 1440 × 900 pixels and a refresh rate of 60 Hz. The left and right arrow keys of the keyboard were used as response keys. The background luminance of the screen was a mid-level gray (*M* ± SEM = 10.14 ± 0.3 cd/m^2^, measured at a viewing distance of 60 cm with a Digital Luminosity Meter HER-140).

Two types of stimuli were presented, differing in their ease of recognition: familiar stimuli (simple geometric figures) and novel stimuli (abstract irregular patterns). Perceptual familiarity, as in the case of the geometric figures, facilitates highly detailed visual representations and enhances expectation effects during visual processing (Utzerath et al., 2017). The abstract irregular patterns used as novel stimuli were unpredictable and difficult to categorize. Therefore, novel stimuli were also more complex than familiar stimuli. All the stimuli spanned approximately 8.57° of visual angle (about 9 cm at a viewing distance of 60 cm) and were presented in the center of the screen. The familiar stimuli were white (M ± SEM = 12.33 ± 0.2 cd/m^2^) and novel stimuli were composed of different grayscale combinations (M ± SEM = 8.91 ± 0.3 cd/m^2^). Novel stimuli were generated using a program written in the Java language (version 1.7.0_75), which given a field of action (stimulus size) created layers of irregular figures generated by the use of multiple forms, including Bézier curves and straight lines. Each of these forms was assigned a random position in the field of action, ensuring the generation of substantially different abstract irregular patterns.

#### Design

In all the experimental conditions, each trial consisted of a train of five stimuli. The first four standard repetitive stimuli were each presented for a constant duration of 500 ms and the fifth and target stimulus was presented with different comparative durations. A non-adaptive psychophysical procedure was used to determine the comparative durations of the target stimuli. The two extreme comparative durations (c1 and c9) were selected in such a way that covered the full range of the psychometric function from 0 to 1, and the other comparative durations were predetermined around the threshold region using Weber fractions (Rammsayer, 2010). In human timing performance, the obtained Weber fractions are frequently around 0.1–0.15, or even lower (for review, see Wearden and Lejeune, 2008). Using a 0.1 Weber fraction and a standard duration of 500 ms, c1 was selected as 500^∗^(1 - 4^∗^0.1) and c9 as 500^∗^(1 + 4^∗^0.1), resulting in c1 = 300 and c9 = 700. Starting from c1, each level should increase by 50 ms to cover a range of 700 ms. Therefore, target stimulus durations were distributed in a symmetrical manner around the standard duration: 300, 350, 400, 450, 500, 550, 600, 650, or 700 ms. The fifth stimulus in the sequence was always the target stimulus in the task.

Using the findings of Dyjas et al. (2012) as reference, the reminder task was used, where the standard duration always preceded the comparative duration. This was done to control the Type B order effect in temporal discrimination sensitivity, which occurs when modifying the order of presentation of the standard and target stimuli. Additionally, this ensured avoiding sequential effects, conditioned to the preceding trial, in the measured point of subjective equality (PSE). This is one of the main reasons for using a fixed position of the target stimulus and for not presenting any additional standard stimuli after the target.

Each experimental session consisted of 90 trials divided into ten blocks of nine trials. Each trial, in all four experimental conditions, was structured as follows. A red fixation cross spanning a visual angle of 0.38° appeared in the center of the screen. After 800 ms, the first standard stimulus appeared, followed by the rest of the repetitive standards. Subsequently, the target stimulus was presented for one of the randomly selected durations mentioned above. Each duration had 1/9 probability of being selected, without replacement, for each target stimulus trial within each block of nine trials. Hence, the target stimulus was presented with each duration exactly once in each block. The ISI had a fixed duration of 300 ms. The fixation cross remained visible along with the sequence of stimuli and disappeared when presentation of the target stimulus ended and the participant’s response was registered. Participants each completed one practice block and nine experimental blocks. From the point of view of the participant, the practice block was indistinguishable from the experimental blocks; however, these data were excluded from the analysis. In total, each participant completed 324 experimental trials (81 trials per condition) and 36 practice trials (9 trials per condition).

**FIGURE 1:**
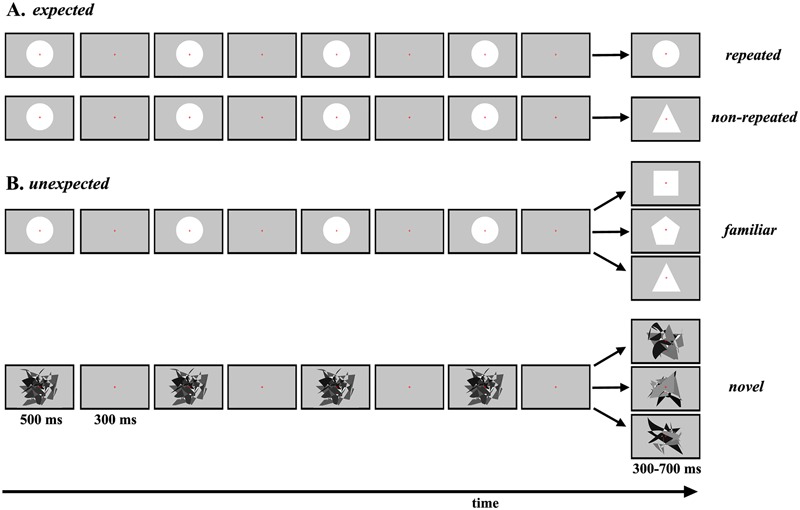
Schematic representation of each experimental condition. **(A)** The sequence of repeated standard stimuli and the target stimulus presented in each trial in the *expected repeated* and *expected non-repeated* conditions. **(B)** The sequence of repeated standard stimuli and the target stimulus presented on three example trials in the *unexpected familiar* and *unexpected novel* conditions.

Four experimental conditions were designed and labeled according to the physical characteristics of the target stimulus: *expected repeated, expected non-repeated, unexpected familiar* and *unexpected novel* (Figure 1). In the conditions with expected target stimuli (Figure 1A), all standard and target stimuli were familiar geometric figures. The manipulation of expectations and salience of the target stimulus was based on stimulus repetition (making the target less salient) and a non-repeated stimulus that was nonetheless perfectly predictable (making the target more salient). Circles were repeatedly presented as standard stimuli, but depending on the experimental condition, the target stimulus was either also a circle *(expected repeated*) or a triangle (*expected non-repeated*). Furthermore, unlike Cai et al. (2015), to induce 100% certainty about the physical characteristics of the target stimulus, only one experimental condition was presented within a session.

In the conditions with unexpected target stimuli (Figure 1B), manipulation of expectations and salience was based on the presentation of different familiar target stimuli (less salient) and novel target stimuli (more salient). In the *unexpected familiar* condition, circles were repeatedly presented as standard stimuli, and one of nine different geometric figures was presented randomly as the target stimulus. Each of the nine geometric figures was presented exactly during a block. In the *unexpected novel* condition, the same abstract irregular pattern was repeatedly presented on all trials. Here, it is essential to highlight that a circle was not used for standard stimuli, to avoid any contextual novelty effects (Schomaker, 2015). For target stimuli, one of 90 different abstract irregular patterns was presented on each trial, so that these patterns were never repeated during the experiment.

#### Procedure

The experiment was run in a silent, dimly illuminated room. Participants were randomly assigned to four different orders of experimental conditions using a balanced Latin Square. They completed one experimental session on each of four consecutive days, each of which corresponded to one experimental condition. Participants received written and verbal instructions before the experiment started and were instructed to make a judgment about whether the duration of the last stimulus in each sequence was shorter or longer than the standard stimulus duration (comparative judgment task). Additionally, they were informed that the first four stimuli in each sequence had a constant duration and that only the duration of the fifth (target) stimulus varied. Participants were instructed to look at the fixation point and were advised that the task was difficult and they would have to respond in accordance with their subjective impression.

On each trial, the participant sequentially viewed five stimuli presented in the center of the screen, one stimulus at a time, and judged the last of these. Participants were cued to respond 300 ms after presentation of the fifth stimulus in order to avoid action preparation effects (Hagura et al., 2012). They pressed the left or the right arrow key to indicate whether its duration had been shorter or longer than that of the first four stimuli. After the participant’s response, the next trial began 800 ms later. No feedback was provided. The first block was always the practice block. Breaks were integrated between blocks to give participants the opportunity to relax and refocus. All breaks were terminated by the participant via spacebar keypress.

#### Data Analysis

The relative duration distortion (RDD) was the main dependent variable (see the equation below). The RDD represents the magnitude of the OE in relation to the PSE. The PSE represents the target stimulus duration that was perceived as being equal to the standard duration. Once the PSE was obtained, the RDD of the target stimulus was calculated as by Cai et al. (2015) using the following equation:

$$RDD=\frac{t_{\text{sta}}-PSE}{PSE}$$

where *t*_sta_ is the duration of the standard stimulus. For example, an RDD of 0.09 means that the target stimulus was judged to have been presented for 9% longer than the duration of the standard stimuli. Logistic functions were fitted to the data for each experimental condition for each participant in order to compute the PSE. A smaller PSE indicates that the participant tends to perceive the target stimulus as lasting longer than the standard. The fits of the logistic functions were computed according to the following equation (Beckmann and Young, 2009):

$$y=\frac{1}{1+e^{-(s^{*}(t-b))}}$$

where *t* is the duration of the target stimulus, *s* is the sensitivity parameter responsible for the slope of the function, and *b* is a bias parameter representing the PSE (responsible for left or right function shifts). The non-linear least-squares function (Bates and Watts, 1988) within the *nls* package of the statistical software application R Development Core Team (2008) was used to estimate the parameters. The difference limen (DL) was computed based on the fit of each logistic functions as a measure of temporal discrimination sensitivity. The DL is half the difference of the duration at which 75% of the targets were judged “longer,” minus the duration at which 25% of the targets were judged “longer.” A larger DL indicates poorer temporal discrimination.

### Results

**FIGURE 2:**
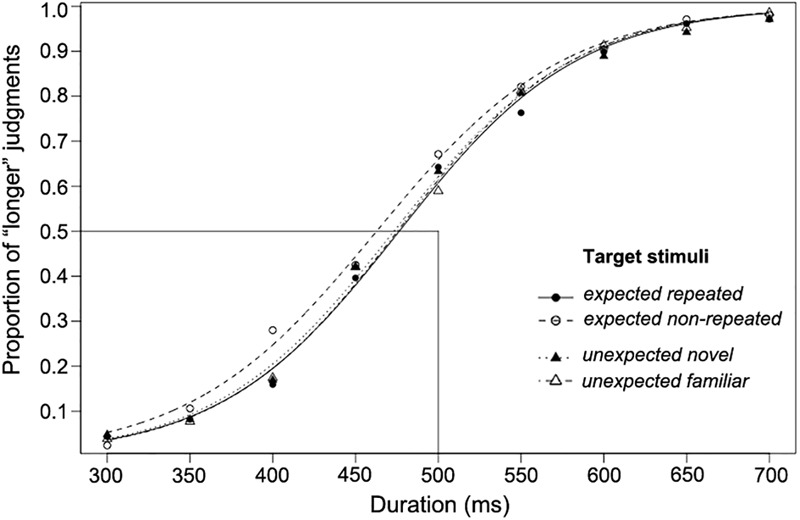
The proportion of “longer” judgments made for each duration and psychometric curves fitted for expected and unexpected target stimulus conditions over 23 participants (Experiment 1).

The proportion of participants’ “longer” judgments for target stimuli of each comparison duration, together with the logistic functions fitted for each of the four experimental conditions, are shown in Figure 2. As this figure shows, the fitted psychometric functions are shifted to the left, indicating that a shorter duration was perceived to be the same length as the standard duration (500 ms). The functions shown are only for illustration; individual PSE, RDD, and DL values entered into the following analyses were obtained from individually fitted psychometric functions. All individual data of Experiment 1 are reported in Supplementary Table 1.

To compute the magnitude of the OE in each experimental condition and to perform the planned orthogonal contrasts based on the experimental hypotheses, the PSE data were used to calculate the RDD. First, the confidence intervals of the PSE were calculated to evaluate whether the geometric or the arithmetic means better represented the data for each experimental condition. As shown in Table 1, both the arithmetic mean and the geometric mean fell within the confidence intervals. The arithmetic mean of the PSE obtained individually for each participant was used to calculate the RDD.

**Table 1:** 95% confidence intervals for the point of subjective equality of duration (ms) in each experimental condition and the respective arithmetic and geometric mean (Experiment 1).

Experimental condition	95% confidence interval		Point of subjective equality	
	Lower	Upper	Arithmetic mean	Geometric mean
Expected repeated	469	484	477	474
Expected non-repeated	456	469	459	456
Unexpected novel	466	480	472	468
Unexpected familiar	469	481	478	475

**FIGURE 3:**
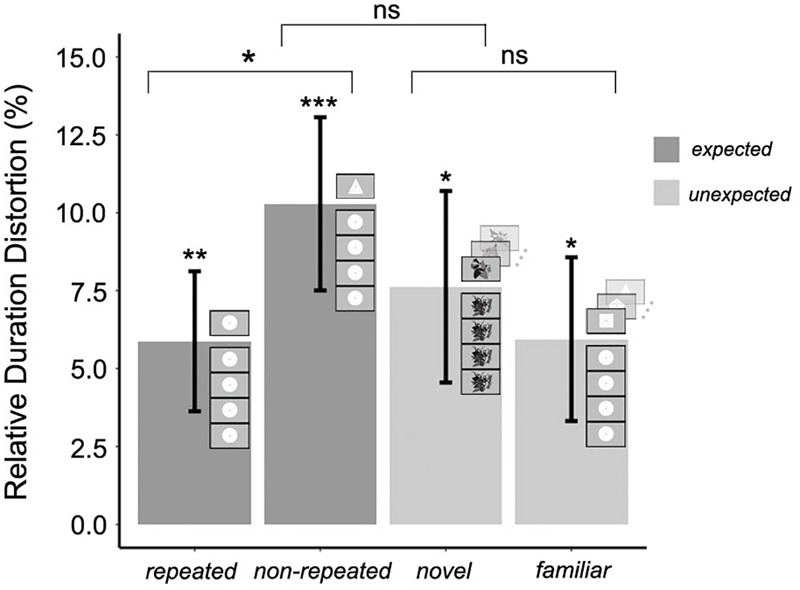
Mean relative duration distortion for expected and unexpected target stimulus conditions in Experiment 1; error bars represent the standard error of the mean. The significance of the magnitude of the oddball effect in each experimental condition (Bonferroni correction for multiple one-tailed *t*-tests was used; *p*-value was significant if *p* < 0.01) and the significance of the three orthogonal planned contrasts (no need for *p*-value correction; *p*-value was significant if *p* < 0.05) between and within expected and unexpected target stimulus conditions is shown (^∗^*p* < 0.05; ^∗∗^*p* < 0.01; ^∗∗∗^*p* < 0.001).

The mean RDD was calculated for each experimental condition. The duration of the target stimulus was judged to be 5.8% (±SEM 2.2%) longer than the standard duration in the *expected repeated* condition, and 10.27% (±SEM 2.7%) longer in the *expected non-repeated* condition. Moreover, the duration of the target stimulus was judged to be 7.6% (±SEM 3.0%) longer than the standard duration in the *unexpected novel* condition, and 5.9% (±SEM 2.6%) longer in the *unexpected familiar* condition. To test whether each of these RDDs was significantly greater than zero, one-tailed *t*-tests were conducted, using the Bonferroni correction for multiple comparisons; therefore, differences were considered significant if *p* < 0.01. The RDD was significantly greater than zero in all cases (*t*_22_ = 2.616, *p* = 0.007; *t*_22_ = 3.699, *p* < 0.001; *t*_22_ = 2.481, *p* = 0.01; *t*_22_ = 2.267, *p* = 0.01, respectively), confirming that the duration of the target stimulus was overestimated in all experimental conditions. The significance of the OE for each experimental condition is shown in Figure 3. It must be noted that the term OE is inadequate for the *expected repeated* condition as the term contains the word “oddball.” However, the term OE in this experimental condition will be used for ease of understanding and coherence throughout the text.

Three orthogonal planned contrasts were then performed (Figure 3). Since the planned contrasts were based on specific, independent hypotheses, no adjustment was made to the chosen confidence interval of 95% (Quinn and Keough, 2002). In the first contrast, RDDs for expected and unexpected target stimulus conditions were compared. As hypothesized, this contrast revealed no significant difference in the RDDs between these two conditions, *F*(1,66) = 0.726, *p* = 0.39. In the second contrast, RDDs for the *expected repeated* (less salient) and *expected non-repeated* (more salient) conditions were compared. As hypothesized, the saliency of non-repeated target stimuli produced a significantly greater increase in perceived duration compared to that associated with repeated target stimuli, *F*(1,66) = 4.204, *p* = 0.04. However, and contrary to the hypothesis, the third contrast, carried out to compare *unexpected familiar* (less salient) and *unexpected novel* (more salient) conditions, revealed no significant effect of saliency, *F*(1,66) = 0.61, *p* = 0.43. Thus, the saliency of expected target stimuli increased the magnitude of the OE, but saliency did not modulate the perceived duration of unexpected target stimuli.

The effects on DL of expected and unexpected target stimuli were also analyzed. The mean DL was 52.8 ms (±SEM 3.9 ms) in the *expected repeated* condition, 46.1 ms (±SEM 3.8 ms) in the *expected non-repeated* condition, 46.2 ms (±SEM 5.3 ms) in the *unexpected novel* condition, and 42.2 ms (±SEM 3.78 ms) in the *unexpected familiar* condition. A repeated measures analysis of variance (ANOVA) revealed no significant effect of expected or unexpected target stimuli on DL, *F*(3,66) = 1.432, *p* = 0.24, indicating that temporal discrimination sensitivity did not differ between experimental conditions.

Experiment 1 revealed that both expected and unexpected target stimuli induced the same level of increase in perceived duration. Moreover, there was no evidence that expectedness influenced the participants’ temporal discrimination sensitivity. Regarding the expected stimuli conditions, expected target stimuli elicited the OE irrespective of whether the participant’s expectations were for a repeated stimulus or for a non-repeated but expected stimulus. In the *expected repeated* condition, the target stimulus was identical in its physical characteristics to the standard stimuli and still elicited the OE. Furthermore, taking into account the fact that standard stimuli were also expected, this finding supports the suggestion that top-down attention is sometimes a sufficient factor to explain the OE. However, there were significant differences in participants’ overestimation of the duration of expected target stimuli between the *expected repeated* and the *expected non-repeated* conditions. The additional contribution of the expected salient features, in terms of the target’s distinctiveness among the standard stimuli, facilitated its detection and fast extraction of sensory information. These results are in line with the idea that top-down attention is sometimes necessary to explain the OE, and saliency makes an additional contribution to further increase the effect (Lin and Shimojo, 2017).

Concerning the unexpected stimuli conditions, the durations of familiar and novel unexpected target stimuli were also overestimated, replicating previous findings on the OE. Although the novel stimuli were particularly salient, there were no significant differences between the *unexpected novel* and the *unexpected familiar* conditions. This finding is similar to those of previous studies about perceived duration being overestimated to the same extent, irrespective of the salient features of unexpected target stimuli (Pariyadath and Eagleman, 2007; Schindel et al., 2011). One possibility is that the complex abstract patterns used as novel stimuli required more processing time for the extraction of perceptual information to further increase the magnitude of the OE.

Taken together, these findings are consistent with Horstmann and Herwig’s (2016) suggestion that task-driven and salience-driven perceptual information is prioritized during allocation of top-down attention. Top-down, task-driven information is quickly processed, and during a later phase, the exogenous capture of attention follows; and as a consequence, the latter requires a longer stimulus presentation (Ansorge et al., 2010). Therefore, a different pattern of findings in regard to the overestimation of the durations of expected and unexpected target stimuli that are more or less salient should be observed as result of modifying the temporal scale of stimulus presentation. To test this hypothesis and the scalar properties of timing, in Experiment 2, Experiment 1 was replicated with a long temporal scale of stimulus presentation (1,000 ms).

## Experiment 2

The findings of Experiment 1 suggested that both expected and unexpected target stimuli elicited an OE when using a short temporal scale of stimuli presentation. Saliency modulated the magnitude of the effect, but only for expected target stimuli. These findings suggest several hypotheses for further testing. First, if top-down attention is the main underlying factor in the OE, increasing the temporal scale of stimulus presentation should elicit the same pattern of results regarding overestimation of the duration of expected and unexpected target stimuli. Second, a longer perceptual processing time might diminish the fast detection and processing advantages associated with salient expected target stimuli. Third, if unexpected salient features of target stimuli need more processing time for more perceptual information to be extracted, using a longer temporal scale for stimulus presentation should further increase the magnitude of the OE. Therefore, in Experiment 2, the temporal scale of stimulus presentation was doubled to test these hypotheses.

### Materials and Methods

#### Participants

A new sample of thirty members of the Biotechnology Institute of the National Autonomous University of Mexico participated in the experiment. Of them, four were excluded from the sample because they had missed several sessions. The final sample size was 26 participants (16 women, aged 27–66 years, *M* = 48.7 years). Three participants reported being left-handed. All of them had normal or corrected-to-normal visual acuity. As in Experiment 1, each experimental session lasted approximately 15 min, and participants completed one session on each of four consecutive days. As in Experiment 1, all participants were informed that the experiment was about time perception, but remained naive about the experimental hypotheses. All participants provided informed consent before participating. This study was approved and conducted based on the same ethical principles as applied in Experiment 1.

#### Apparatus, Stimuli, Procedure, Design, and Data Analysis

Except for the duration of the standard and target stimuli, Experiment 2 was identical to Experiment 1. In Experiment 2, a 1,000 ms long standard duration was used in place of the 500 ms short duration of Experiment 1’s standard stimuli. Target stimulus durations were distributed symmetrically around the long standard duration: 600, 700, 800, 900, 1,000, 1,100, 1,200, 1,300, or 1,400 ms.

### Results

**FIGURE 4:**
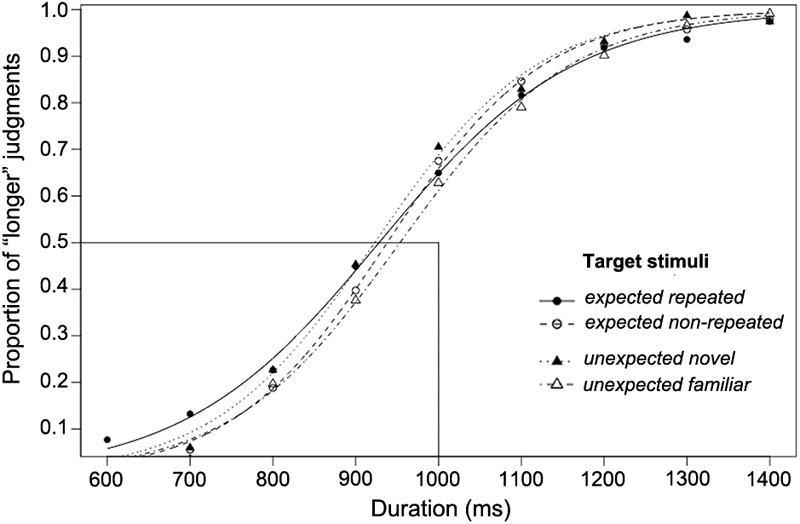
The proportion of “longer” judgments made for each duration and psychometric curves fitted for expected and unexpected target stimulus conditions over 26 participants using a long standard duration (1,000 ms; Experiment 2).

The proportion of participants’ “longer” judgments for the target stimuli of each comparison duration, together with the logistic functions fitted for each of the four experimental conditions, are shown in Figure 4 (these functions are only for illustration; the individual fitted psychometric function for each participant was used in the data analysis). All individual data of Experiment 2 are reported in Supplementary Table 2. The psychometric functions fitted for all experimental conditions were shifted to the left, indicating that the OE occurred, as the PSE fell at a shorter duration than the standard duration (1,000 ms).

**FIGURE 5:**
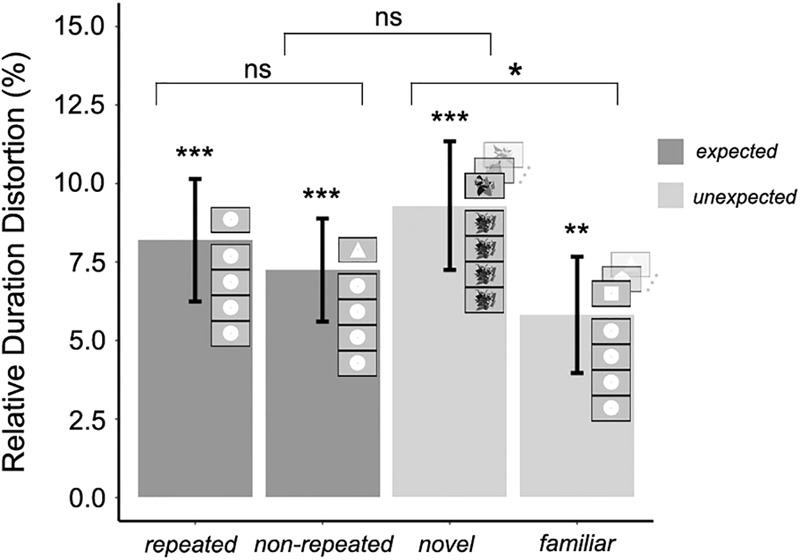
Mean relative duration distortion for expected and unexpected target stimuli with a long timescale of stimuli presentation (Experiment 2); error bars represent the standard error of the mean. The significance of the magnitude of the oddball effect in each experimental condition (Bonferroni correction for multiple one-tailed *t*-tests was used; *p*-value was significant if *p* < 0.01) and the significance of the three orthogonal planned contrasts (no need for *p*-value correction; *p*-value was significant if *p* < 0.05) between and within expected and unexpected target stimulus conditions is shown (^∗^*p* < 0.05; ^∗∗^*p* < 0.01; ^∗∗∗^*p* < 0.001).

Relative duration distortions were computed in the same way as in Experiment 1 in order to measure the magnitude of the OE in each experimental condition and to perform the planned orthogonal contrasts. Table 2 shows that both the arithmetic and the geometric mean fell within the confidence intervals in all the experimental conditions. The mean RDD was calculated for each experimental condition. The duration of target stimuli was judged to be 8.1% (±SEM 1.9%) longer than the standard duration in the *expected repeated* condition, and 7.2% (±SEM 1.6%) longer in the *expected non-repeated* condition. Furthermore, the duration of target stimuli was judged to be 9.3% (±SEM 2.0%) longer than the standard duration in the *unexpected novel* condition, and 5.8% (±SEM 1.8%) longer in the *unexpected familiar* condition. In every condition, the RDD was significantly greater than zero (one-tailed *t*-tests: *t*_25_ = 4.193, *p* < 0.001; *t*_25_ = 4.401, *p* < 0.001; *t*_25_ = 4.539, *p* < 0.001; *t*_25_ = 3.141, *p* = 0.002, respectively), confirming that there was an OE in all four experimental conditions (Figure 5).

**Table 2:** 95% confidence intervals for the point of subjective equality of duration (ms) in each experimental condition and the respective arithmetic and geometric mean (Experiment 2).

Experimental condition	95% confidence interval		Point of subjective equality	
	Lower	Upper	Arithmetic mean	Geometric mean
Expected repeated	918	938	932	928
Expected non-repeated	929	946	938	935
Unexpected novel	910	935	923	919
Unexpected familiar	941	965	952	945

Three orthogonal planned contrasts were then performed to test the hypotheses regarding the expected and unexpected target stimuli (Figure 5). In the first contrast, RDDs for the expected and unexpected conditions were compared. As in Experiment 1, this contrast revealed no significant difference between these conditions, *F*(1,75) = 0.018, *p* = 0.89. In the second contrast, RDDs for the *expected repeated* condition (less salient) and the *expected non-repeated* condition (more salient) were compared. Contrary to Experiment 1, no significant difference in the RDD due to saliency was found, *F*(1,75) = 0.313, *p* = 0.57, when a longer temporal scale was used for stimulus presentation. However, the third contrast, between the *unexpected familiar* (less salient) and *unexpected novel* (more salient) conditions, revealed a significant effect of saliency in the RDD, *F*(1,75) = 4.184, *p* = 0.043. Therefore, and unlike in Experiment 1, saliency increased the magnitude of the OE in the case of unexpected target stimuli, but did not do so in the case of expected target stimuli.

The effects on DL of expected and unexpected target stimuli were also analyzed. Mean DLs were 115.4 ms (±SEM 15.4 ms) in the *expected repeated* condition, 91.6 ms (±SEM 8.7 ms) in the *expected non-repeated* condition, 87.8 ms (±SEM 5.4 ms) in the *unexpected novel* condition, and 94.4 ms (±SEM 8.7 ms) in the *unexpected familiar* condition. A repeated measures ANOVA revealed no significant effect of target stimulus type on DL, Greenhouse-Geisser adjusted *F*(2.025,50.67) = 2.037, *p* = 0.14. This indicates that, as in Experiment 1, the participants’ temporal discrimination sensitivity did not differ between experimental conditions.

Experiment 2 revealed that using a long temporal scale of stimulus presentation elicited no significant differences in the extent to which the durations of expected and unexpected target stimuli were overestimated, nor in the temporal discrimination sensitivity across experimental conditions, as in Experiment 1. However, contrary to the findings of Experiment 1, saliency did not increase the magnitude of the OE for expected target stimuli, although it did have a significant effect on the OE for unexpected target stimuli. The perceptual advantages conferred by salient expected target stimuli in Experiment 1, which meant that they were quickly detected and processed, producing an increased OE, vanished at the larger temporal scale of stimulus presentation used in Experiment 2. Therefore, it seems that salient expected features of target stimuli only contribute to the modulation of the OE when presented on a short temporal scale, favoring faster detection and perceptual processing. Concerning the unexpected target stimuli conditions, there was a significant difference in magnitude between the OEs elicited by novel target stimuli and by familiar target stimuli. As hypothesized, a longer temporal scale of stimulus presentation allowed the extraction of more perceptual information from the target stimulus, further increasing the magnitude of the OE. Taken together, these findings suggest that the perceived duration of a stimulus depends on the speed of perceptual information processing and consequently the amount of such information processed over a specific temporal scale.

#### Scalar Properties of Timing

The temporal scales of stimulus presentation used in Experiment 1 (500 ms) and Experiment 2 (1,000 ms) were in a constant ratio of 1:2. Hence, it was possible to analyze the scalar properties of timing. With the long temporal scale of stimulus presentation used in Experiment 2, the DL increased, indicating that the participants’ absolute temporal discrimination sensitivity decreased in comparison to Experiment 1. However, to test whether relative temporal discrimination sensitivity remained constant as the judged duration varied, Weber fractions were calculated by dividing the DL by the PSE for each participant’s data obtained in Experiment 1 and Experiment 2. The resulting Weber fractions did not differ between the experiments, indicating that relative temporal discrimination sensitivity remained constant (Table 3). These findings are consistent with the variation in Weber fractions generally obtained from human timing performance, which are frequently around 0.10–0.15, or even lower (for a review, see Wearden and Lejeune, 2008).

**Table 3:** Weber fractions (mean and the standard error of the mean, SEM) for each experimental condition in Experiment 1 and Experiment 2.

Experimental condition	Experiment 1 (500 ms temporal scale)		Experiment 2 (1,000 ms temporal scale)	
	Mean	±SEM	Mean	±SEM
Expected repeated	0.11	0.008	0.12	0.016
Expected non-repeated	0.10	0.009	0.09	0.009
Unexpected novel	0.10	0.012	0.09	0.005
Unexpected familiar	0.08	0.008	0.10	0.009

**FIGURE 6:**
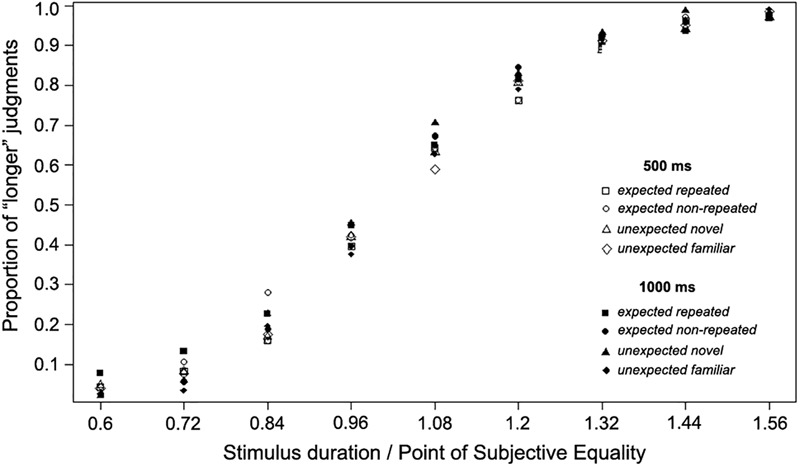
Proportion of “long” judgments made across Experiments 1 and 2, plotted using the same relative scale, normalized for short (500 ms) or long (1,000 ms) temporal scale of stimulus presentation.

To further examine the scalar properties of timing, the superposition method was used. That is, the relative proportions of “longer” judgments made in Experiment 1 and Experiment 2 were plotted using the same relative scale by dividing each of the nine durations used in each case by the arithmetic mean of the PSE, corresponding to each experimental condition (Figure 6). A good superposition of the data was observed, also confirming the scalar properties of timing.

## Discussion

In the experiments described in this article, participants’ expectations and the saliency of target stimuli were manipulated using a temporal oddball task in order to dissociate their specific contribution in eliciting and modulating the OE. Three main findings emerged from the present investigation. First, the engagement of top-down attention to target stimuli, irrespective of their expected or unexpected nature, elicited the OE to the same extent. Therefore, top-down attention allocated to a task-relevant stimulus is a necessary factor in explaining the OE. Moreover, a higher-order repetition effect, in which observers formulate expectations about repetition (Matthews’, 2015; Skylark and Gheorghiu, 2017), only increased perceived duration when top-down attention was allocated to a repeated target stimulus. In other words, an expected task-relevant stimulus elicited an OE although it was indistinguishable in its physical features from the equally expected, repeated standard stimuli. Second, a small but significant contribution from saliency was observed with salient expected and unexpected target stimuli, but this effect was dependent on the temporal scale of stimulus presentation. In Experiment 1, in which a short temporal scale of stimulus presentation (500 ms) was used, salient expected target stimuli elicited a further increase in the OE, but saliency did not affect the perceived duration of unexpected target stimuli. On the other hand, in Experiment 2, in which a long temporal scale of stimulus presentation was used (1,000 ms), salient unexpected target stimuli elicited a further increase in the OE, but in this case, the additional contribution of saliency to the OE elicited by expected target stimuli was eliminated. Third, the scalar properties of timing were investigated, suggesting that a similar temporal mechanism is engaged to perform comparative judgments in a temporal oddball task even when the stimuli are presented on different temporal scales. The implications of these findings are discussed below in relation to the assumptions made by different theoretical accounts.

The attentional account of the OE (Tse et al., 2004) suggests that the engagement of attention by an unexpected stimulus increases perceptual information processing, and as a result, the perceived duration of the stimulus is increased. This account suggests that variables that increase the rate of perceptual information processing of a stimulus, such as its oddness or unexpectedness, or presence of salient physical features, should enhance the magnitude of the OE. In the present experiments, the OE was elicited to the same extent by expected and unexpected features of the target stimuli. This finding suggests that the top-down attention allocated to a task-relevant stimulus increases its perceived duration regardless of the unexpectedness of the stimulus. Saliency was an additional contributing factor that modulated the magnitude of the OE. However, the presence of top-down attention to target stimuli was sufficient to elicit the OE. For instance, in the *expected repeated* experimental conditions described in this article, a repeated target stimulus, identical in its physical features to the standard stimuli, also elicited the OE. Even when the observer attends to the same location and perceives an identical stimulus, top-down attentional control enhances relevant stimulus components in accordance with the perceptual task at hand (Gilbert and Li, 2013). In fact, the arousal account of the OE could not explain these results, leaving top-down attention as the main mechanism that increases the rate of information processing and therefore the perceived duration of the stimulus.

The predictive coding account of the OE suggests that repeated or expected stimuli elicit a reduction in their perceived duration, arising from the fact that such stimuli are more efficiently coded (Pariyadath and Eagleman, 2012). If efficient coding of a stimulus reduces its perceived duration, in the present experiments, the OE would not have been elicited when expected target stimuli were presented. Importantly, significant differences between expected and unexpected target stimuli were not observed. Moreover, the predictive coding account suggests that the magnitude of the OE increases monotonically with the degree of discrepancy between the standard stimuli and the oddball as a result of the size of the elicited prediction error (Schindel et al., 2011; Pariyadath and Eagleman, 2012). This assumption can be explained with reference to the additional contribution of saliency in modulating the magnitude of the OE, which was small but significant. The degree of the discrepancy between the target and standard stimuli, which refers to the number of features of the target stimulus that change in relation to the repeated standards, governs the extent to which the target is more salient, and thus the extent to which the magnitude of the OE is further increased. Therefore, our results are in conflict with the idea that perceived duration correlates with the neural response evoked by a stimulus. In further conflict with this view, additional empirical evidence has demonstrated that the duration of a target stimulus is overestimated even if it is presented within random sequences of standard stimuli (Cai et al., 2015; Lin and Shimojo, 2017). Indeed, unexpected stimuli evoke a neural response of greater magnitude irrespective of the relevance of the stimulus to the task (Courchesne et al., 1975; Kok et al., 2012). Together, these findings suggest that how efficiently a stimulus is coded does not play a critical role in determining its perceived duration.

As mentioned in the introduction, the expectation-based accounts share the assumption that an oddball stimulus is perceived as lasting longer because it is unexpected and repeated stimuli are perceived as shorter in duration because they are expected. Contrary to what these expectation-based accounts suggest, we interpret the OE in terms of the influence of top-down attention, consistent with previous research that has ruled out unpredictability as the main explanatory factor in the OE (van Wassenhove et al., 2008; New and Scholl, 2009; Lin and Shimojo, 2017). Moreover, the present findings are consistent with Lin and Shimojo’s (2017) findings on the underlying factors in the OE. Top-down attention is an inherent explanatory factor in the OE, sometimes sufficient and sometimes necessary. In the latter case, our results suggest that the additional contribution of saliency explains, to some degree, how the magnitude of the effect is modulated. The influence of the saliency of expected and unexpected target stimuli on their perceived durations was dependent on the temporal scale of stimulus presentation. Therefore, we suggest that the amount of perceptual information extracted within a specific timeframe might be relevant to determine the extent to which the duration of target stimuli is overestimated. This is the first approach to start making conclusions about this concern.

Experiment 1 shed light on the contribution of saliency in guiding top-down attention to quickly detect and process the expected features of a target stimulus, thereby further increasing the OE. However, in Experiment 2, in which a long temporal scale of stimuli presentation was used, no significant differences were observed between expected target stimuli with less salient features and those with more salient features. This finding suggests that the perceptual advantages conferred by saliency in quickly detecting and extracting sensory information from expected target stimuli are diminished when more time is available to detect a task-relevant stimulus. Concerning unexpected target stimuli, the opposite pattern of results in Experiments 1 and 2 was observed in relation to the contribution of saliency at different temporal scales of stimulus presentation. This pattern can be accounted for by the fact that top-down processing of task-driven information is prioritized (Bacon and Egeth, 1997; Einhäuser et al., 2008; Horstmann and Herwig’s, 2016), followed by exogenous attention at a later stage, requiring a longer stimulus presentation (Ansorge et al., 2010). Unlike familiar stimuli, novel stimuli demand more perceptual processing resources (Kahneman, 1973; Kok and de Jong, 1980; Segaert et al., 2013; Reggev et al., 2016), specifically when they are judged to be task-relevant (Chong et al., 2008). Additionally, novel stimuli require a continued processing after initial categorization, which is associated with the gradual build-up of representations of perceptual stimulus characteristics (Shaffer and Shiffrin, 1972; Kok and de Jong, 1980; Loftus et al., 1983; Loftus, 1985). Taken together, these findings suggest that perceived duration might correlate with the amount of relevant perceptual information processed in a bottom-up manner, which is driven by top-down attentional control.

The present findings can be explained by a broader theoretical framework that has been labeled the *processing principle* (Matthews and Gheorghiu, 2016; Matthews and Meck, 2016). Under this framework, the strength of the percept, which concept refers to the ease with which information can be extracted from the stimulus, determines perceived duration. The strength of the percept depends on the interplay of top-down and bottom-up processes. Therefore, it has been suggested that: (a) mere repetition of a stimulus reduces the strength of the sensory signal associated with it, via adaptation, reducing its perceived duration; (b) novel or unexpected stimuli are associated with a boost to the incoming signal because of their attentional saliency, increasing their perceived duration; and (c) fulfilled expectations about a stimulus lead to top-down enhancement of processing of the anticipated, relevant features, increasing its perceived duration. Although the current study supports the processing principle framework, it provides some constraints.

With respect to the effects of stimulus saliency suggested by the processing principle, repeating the standard stimuli prior to the target stimulus should enhance the OE when this repetition helps the target in “standing out” from the background context (Matthews and Meck, 2016). However, our results suggest that the additional contribution of expected and unexpected salient features of the target stimulus in enhancing the OE depends on the temporal scale of stimulus presentation. Further research is necessary to determine the effect of stimulus saliency on perceived duration. As observed in Experiment 1, it has been reported that, when a short temporal scale of stimuli presentation is used (500 ms), salient expected target stimuli further increase the OE (van Wassenhove et al., 2008), but when target stimuli are unexpected, the salient features have no effect on modulating the magnitude of the OE (Pariyadath and Eagleman, 2007; Schindel et al., 2011). Therefore, it would be useful to test different temporal scales of stimuli presentation using different types of unexpected and expected salient target stimuli (e.g., stimuli with biological significance, as in the case of looming stimuli or threatening faces).

Our results suggest a particular concern regarding the processing principle’s interpretation of the overestimation in duration of expected stimuli. This idea is mainly based on the opposing effects on perceived duration of stimuli repetition, where a first-order repetition effect reduces perceived duration due to a low-level adaptation process, and a high-order repetition effect increases perceived duration because of expectations about repetition (Matthews’, 2015). In the present experiments, the duration of an expected target stimulus was overestimated in comparison with those of standard stimuli, although the latter were also expected. In line with our findings in the *expected repeated* condition, Cai et al. (2015) in their Experiment 2, found that the underestimation of the duration of the last and target stimulus of the sequence diminished when its repetition became expected. However, van Wassenhove et al. (2008), in their visual control conditions, in which the target was identical to the standards, reported an underestimation of the target stimulus duration; however, this was also expected in its physical characteristics. In the study of van Wassenhove et al. (2008), the ISI was pseudo-randomly chosen from 750 ms to 950 ms in steps of 20 ms. On the other hand, a fixed ISI was used in the study of Cai et al. (2015), as was the case in our experimental design. Thus, the observers’ expectations about the target’s onset, which facilitate top-down attention, might explain why an expected repetition of the target stimulus was overestimated in duration. Top-down attentional mechanisms facilitate shape-specific anticipations of the expected features of a target stimulus, which in turn facilitate subsequent detection and processing of the stimulus (Stokes et al., 2009; Ansorge et al., 2010; Summerfield and de Lange, 2014; Ekman et al., 2017). Therefore, our experiments shed light on the fact that when the target stimulus position is fixed and temporally expected, fulfilled expectations increase the perceived duration of a stimulus as part of an interaction with top-down attention allocated to a task-relevant stimulus. Under this view, and contrary to the processing principle, in a temporal oddball task, repeated expected standard stimuli should not be associated with an increase in their perceived duration. This hypothesis needs further testing regarding the opposing effects of expectations on perceived duration when a temporally expected stimulus is or is not task-relevant.

From a mechanistic standpoint, this interpretation could be explained by the pacemaker-accumulator model of timing (Tse et al., 2004; Seifried and Ulrich, 2011). Traditionally, the pacemaker-accumulator model suggests that temporal information is lost when attention is distracted from the temporal information pertaining to an event. However, “the processing of durational information may also get a boost when one attends to a stimulus” (Tse et al., 2004, p. 1186). Under this view, when an observer attends to a stimulus, the accumulated pulses emitted by the pacemaker measure the amount of information processed in order to calculate its duration. Our findings suggest that the internal clock accelerates when top-down attention is allocated to a target stimulus, increasing the rate of information processed bottom-up, meaning that more pulses are accumulated. Thus, the magnitude of the overestimation of the duration of a target stimulus seems to depend on the amount of perceptual information processed at a specific temporal scale. Regarding the presence of salient expected features in a target stimulus, we suggest that because they are anticipated and easily detected, these features cause the internal clock to accelerate quickly, producing a further increase in the magnitude of the OE when a short temporal scale is used. On the other hand, salient unexpected features of a target stimulus need more processing time to trigger this acceleration of the internal clock, producing a further increase in the magnitude of the OE only when a long temporal scale is used. Furthermore, the temporal discrimination sensitivity (DL) should remain unchanged by the allocation of attention, even if the rate of information processing increases (Tse et al., 2004). Accordingly, in our Experiment 1, as in Experiment 2, there were no significant differences in the observers’ temporal discrimination sensitivity between expected, unexpected, and more or less salient target stimuli. Considering the findings of Dyjas et al. (2012) as reference, given that the DL did not differ among our experimental conditions, the differences in the magnitude of the OE observed in some paired comparisons should have a perceptual origin rather than decisional biases caused by sequential effects. Additionally, the scalar properties of timing were observed, favoring the pacemaker-accumulator as the underlying timing mechanism. “The scalar property can be seen as a commonality between time judgments and other cognitive processes, which is encouraging for the development of integrated accounts” (Matthews and Meck, 2016, p. 869).

The current findings do not contradict the proposal of the predictive brain hypothesis. Clark (2013) proposes a “predictive processing” account, referring to perceptual information processing based on predictive coding strategies. Predictive processing leaves unspecified the details of the neural implementation, aiming instead to identify common perceptual processing principles (Clark, 2016). Predictive processing suggests that the brain combines previously stored knowledge (internal models) with incoming sensory information in order to generate the hypothesis that best matches upcoming sensory information during a task embedded in a particular context (Lupyan and Clark, 2015). Unexpected deviations from what is predicted produce prediction errors, which drive further processing to refine predictions until the actual representation matches the sensory signal (Clark, 2015). In contrast, fulfilled expectations about the anticipated sensory information reduce prediction error. However, fulfilled expectations can also induce a top-down enhancement of relevant predicted incoming sensory information (Friston, 2005; Clark, 2013, 2016). Under this framework, attention modulates the interactions between top-down and bottom-up processing in such a way that the enhancement of sensory inputs is facilitated by a top-down attentional mechanism, and controls the influence of prior expectations that are dependent on the task and context (Friston, 2009; Clark, 2013). This attentional mechanism increases the impact on the ongoing processing of task-relevant sensory information (Clark, 2016).

The predictive processing account suggests that expectations guide top-down attention to the most informative and relevant perceptual information for a specific task. Top-down attention allocated to task-relevant expected stimulus features should speed up perceptual processing, but if relevant unexpected features are encountered, these must be processed in depth, requiring more processing time. This predictive processing determines the flow of the bottom-up signal and top-down predictions, favoring the generation of vivid perceptual representations. These ideas are compatible with our suggestion that perceived duration depends on the amount of perceptual information processed in a bottom-up manner, but one that is driven by top-down attentional control to efficiently extract perceptual information relevant to a task.

It must be noted that central processes involving perceptual judgments cannot be understood entirely in terms of sense organs, and therefore, response bias must be considered in any adequate description (Poulton, 1968). The response frequency equalization model of psychophysics suggests a response bias tendency of participants to use available responses with equal frequency (Erlebacher and Sekuler, 1971; Sekuler and Erlebacher, 1971). This response bias mostly affects the judgments of the comparative stimulus that are more similar to the standard, which evoke the uncertain states (Sekuler and Erlebacher, 1971). Thus, psychophysical experiments employing the method of constant stimuli should use preferably comparative stimuli that are distributed in a symmetrical manner around the standard stimulus and with an equal frequency distribution (Parducci and Haugen, 1967; Erlebacher and Sekuler, 1971). However, even if these precautions are taken, given the assumption that the nature of the OE increases participants’ tendency to respond “longer,” the uncertain states might be judged as “shorter” to a greater extent to maintain a balance between their frequency of use. Therefore, it is possible that the blocked design used in the present experiments could have diminished the OE by reducing the shifts in the PSE produced by the response bias tendency. If this is true, in a temporal oddball task, the blocked designs may lower the chance of detecting significant differences between experimental conditions. To the best of our knowledge, no previous study has had the primary research objective of testing the effects of blocked and interleaved designs in modulating the magnitude of the OE. Nevertheless, the RDDs reported in the present experiments (ranging from 5.8 to 10.27%) are consistent with previous findings in which repeated standard stimuli were used, but the experimental conditions were interleaved (e.g., Schindel et al., 2011; Cai et al., 2015; Lin and Shimojo, 2017). However, further research is needed with respect to the response bias tendency under blocked or interleaved designs when using the method of constant stimuli to analyze its effects on the OE.

Finally, action preparation effects on perceived duration could be an additional contributing factor for eliciting and modulating the magnitude of the OE. It has been reported that the perceived duration of a visual stimulus is overestimated when it is presented before execution of a voluntary action (Hagura et al., 2012; Iwasaki et al., 2017). The direction and modulation of action preparation effects on perceived duration depend on the presence or absence of sensory input during the period to be judged before the execution of the action (Iwasaki et al., 2017). The overestimation of duration of a stimulus occurs when it is presented in the pre-action period (Hagura et al., 2012; Iwasaki et al., 2017), but if the ISI has to be judged in the pre-action period, its perceived duration is contracted (Morrone et al., 2005; Tomassini et al., 2014; Yokosaka et al., 2015) or there may be no effect on perceived duration (Iwasaki et al., 2017). Thus, it might be possible to consider action preparation effects as an adaptive function, which in the presence of sensory input, boosts the speed of visual processing to maximize opportunities to adjust actions as needed (Hagura et al., 2012; Iwasaki et al., 2017). These findings about action preparation effects are consistent with the assumption that perceived duration correlates with the speed and amount of perceptual information processed.

Of particular interest for the present study, it has been reported that in a detection rate task, visual processing performance increases to a greater extent when an action is involved than when one is only directing top-down attention to the task. This increase in visual processing performance occurs over the final 300 ms before the action is executed (Hagura et al., 2012). In order to avoid action preparation effects, in the experiments reported in this article, participants were cued on each trial to respond after an ISI of 300 ms, which initiated at the off-set of the target stimulus. However, in a classical temporal oddball task, it is still not clear if action preparation effects regarding the target stimulus have an additional contribution to top-down attentional effects on perceived duration even if the action is executed after 300 ms of its presentation. Therefore, further studies are needed to elucidate the contribution of action preparation effects in eliciting or modulating the OE. We suggest three experimental conditions to dissociate the contribution of top-down attentional effects and action preparation effects in a classical temporal oddball task. First, a condition where participants actively respond at the off-set of each stimulus of the sequence. Therefore, standard stimuli and the target stimulus should become relevant for the action preparation task; the target stimulus would also be relevant for the comparison duration task. Second, a condition where the participants actively respond at the off-set of all standard stimuli but not to the target stimulus. Finally, a condition where participants only actively respond at the off-set of the target stimulus presentation. If the OE is still elicited in the first two experimental conditions, then it could be suggested that top-down attention is the main explanatory factor of the OE. Moreover, if the OE significantly increases in the third condition compared to the first two, action preparation effects could be considered as an additional contributing factor that modulates the magnitude of the OE.

## Conclusion

In the present study, participants’ expectations and the saliency of target stimuli were manipulated to disambiguate the specific contributions of these factors in eliciting the OE. The findings indicated that the OE, measured by the RDD, was elicited to the same extent by expected and unexpected target stimuli. The same pattern of results was observed using a short and a long temporal scale of stimulus presentation. This finding is in line with the assumption that top-down attention is the main factor underlying the OE. Moreover, a small but significant difference in the magnitude of the OE was elicited by varying the saliency of target stimuli, but this effect was dependent on the temporal scale of stimulus presentation. Here is suggested that when a short temporal scale was used, the presence of salient expected features in the target stimulus speeded up perceptual processing, perhaps conferring a perceptual processing advantage. On the other hand, when a long temporal scale was used, the presence of salient unexpected features in target stimuli further increased the magnitude of the OE, but eliminated the contribution of the presence of expected salient features. The perceptual advantages of saliency in quickly detecting and extracting sensory information from an expected target stimulus were eliminated when more time was available to detect a task-relevant stimulus. Salient unexpected target stimuli incur perceptual and temporal disadvantages, suggesting that a longer temporal scale of stimulus presentation is required to extract perceptual information relevant to the task in order to increase perceived duration further.

Our results suggest that top-down attention is the primary cognitive mechanism associated with perceived duration, as a result of its role in the perceptual information processing of task-relevant stimuli. Moreover, we suggest that top-down attention to target stimuli, in addition to the salient expected and unexpected features processed within a specific temporal scale, might be the main factors underlying the OE, as in conjunction they determine the amount and the speed of information that can be processed. We believe these findings point to new research directions that warrant further examination. Finally, the present study favors the pacemaker-accumulator model of timing, given the assumption the internal clock accelerates when top-down attention is allocated to the processing of perceptual information that is relevant to a task.

## Data Availability

The raw data supporting the conclusions of this manuscript and the software used to generate novel stimuli will be made available by the authors, without undue reservation, to any qualified researcher.

## Author Contributions

AC conceived the study. AC, FL, and BL contributed to the design of the experiments. AC collected and analyzed the data, and wrote the first draft of the manuscript. All authors contributed to manuscript revision, and read and approved the submitted version.

## Conflict of Interest Statement

The authors declare that the research was conducted in the absence of any commercial or financial relationships that could be construed as a potential conflict of interest.

## References

[B1] Allman M. J. Teki S. Griffiths T. D. Meck W. H. (2014). Properties of the internal clock: first- and second-order principles of subjective time. *Annu. Rev. Psychol.* 65 743–771. 10.1146/annurev-psych-010213-115117 24050187

[B2] Ansorge U. Horstmann G. Scharlau I. (2010). Top-down contingent attentional capture during feed-forward visual processing. *Acta Psychol.* 135 123–126. 10.1016/j.actpsy.2010.05.008 20883842

[B3] Bacon W. F. Egeth H. E. (1997). Goal-directed guidance of attention: evidence from conjunctive visual search. *J. Exp. Psychol. Hum. Percept. Perform.* 23 948–961. 10.1037/0096-1523.23.4.948 9269723

[B4] Bar M. (2007). The proactive brain: using analogies and associations to generate predictions. *Trends Cogn. Sci.* 11 280–289. 10.1016/j.tics.2007.05.005 17548232

[B5] Bates D. M. Watts D. G. (eds) (1988). *Nonlinear Regression Analysis and Its Applications. Wiley Series in Probability and Statistics.* New York, NY: Wiley 10.1002/9780470316757

[B6] Beckmann J. S. Young M. E. (2009). Stimulus dynamics and temporal discrimination: implications for pacemakers. *J. Exp. Psychol. Anim. Behav. Process.* 35 525–537. 10.1037/a0015891 19839705

[B7] Bernstein I. H. Reese C. (1965). Behavioral hypotheses and choice reaction time. *Psychon. Sci.* 3 259–260. 10.3758/bf03343125

[B8] Birngruber T. Schröter H. Schütt E. Ulrich R. (2018). Stimulus expectation prolongs rather than shortens perceived duration: evidence from self-generated expectations. *J. Exp. Psychol. Hum. Percept. Perform.* 44 117–127. 10.1037/xhp0000433 28481566

[B9] Birngruber T. Schröter H. Ulrich R. (2014). Duration perception of visual and auditory oddball stimuli: does judgment task modulate the temporal oddball effect? *Atten. Percept. Psychophys.* 76 814–828. 10.3758/s13414-013-0602-2 24435899

[B10] Buhusi C. V. Meck W. H. (2005). What makes us tick? Functional and neural mechanisms of interval timing. *Nat. Rev. Neurosci.* 6 755–765. 10.1038/nrn1764 16163383

[B11] Buonomano D. (2017). *Your Brain Is a Time Machine: The Neuroscience and Physics of Time.* New York, NY: W. W. Norton & Company.

[B12] Cai M. B. Eagleman D. M. Ma W. J. (2015). Perceived duration is reduced by repetition but not by high-level expectation. *J. Vis.* 15:19. 10.1167/15.13.19 26401626 PMC5833204

[B13] Chong H. Riis J. L. McGinnis S. M. Williams D. M. Holcomb P. J. Daffner K. R. (2008). To ignore or explore: top-down modulation of novelty processing. *J. Cogn. Neurosci.* 20 120–134. 10.1162/jocn.2008.20003 17919081

[B14] Church R. M. (1984). Properties of the internal clock. *Ann. N. Y. Acad. Sci.* 423 566–582. 10.1111/j.1749-6632.1984.tb23459.x 6588815

[B15] Church R. M. (2003). “A concise introduction to scalar timing theory,” in *Functional and Neural Mechanisms of Interval Timing* ed. Meck W. H. (Boca Raton, FL: CRC Press) 3–22. 10.1201/9780203009574.sec1

[B16] Clark A. (2013). Whatever next? Predictive brains, situated agents, and the future of cognitive science. *Behav. Brain Sci.* 36 181–204. 10.1017/S0140525X12000477 23663408

[B17] Clark A. (2015). “Embodied prediction,” in *Open MIND* eds Metzinger T. Windt J. M. (Frankfurt am Main: MIND Group) 1–21. 10.15502/9783958570115

[B18] Clark A. (2016). *Surfing Uncertainty: Prediction, Action, and the Embodied Mind.* Oxford: Oxford University Press 10.1017/s0012217317000270

[B19] Courchesne E. Hillyard S. A. Galambos R. (1975). Stimulus novelty, task relevance and the visual evoked potential in man. *Electroencephalogr. Clin. Neurophysiol.* 39 131–143. 10.1016/0013-4694(75)90003-6 50210

[B20] Dyjas O. Bausenhart K. M. Ulrich R. (2012). Trial-by-trial updating of an internal reference in discrimination tasks: evidence from effects of stimulus order and trial sequence. *Atten. Percept. Psychophys.* 74 1819–1841. 10.3758/s13414-012-0362-4 23055085

[B21] Eagleman D. M. (2008). Human time perception and its illusions. *Curr. Opin. Neurobiol.* 18 131–136. 10.1016/j.conb.2008.06.002 18639634 PMC2866156

[B22] Eagleman D. M. Pariyadath V. (2009). Is subjective duration a signature of coding efficiency? *Philos. Trans. R. Soc. Lond. B Biol. Sci.* 364 1841–1851. 10.1098/rstb.2009.0026 19487187 PMC2685825

[B23] Einhäuser W. Rutishauser U. Koch C. (2008). Task-demands can immediately reverse the effects of sensory-driven saliency in complex visual stimuli. *J. Vis.* 8:2. 10.1167/8.2.2 18318628

[B24] Ekman M. Kok P. de Lange F. P. (2017). Time-compressed preplay of anticipated events in human primary visual cortex. *Nat. Commun.* 8:15276. 10.1038/ncomms15276 28534870 PMC5457495

[B25] Erlebacher A. Sekuler R. (1971). Response frequency equalization: a bias model for psychophysics. *Percept. Psychophys.* 9 315–320. 10.3758/bf03212657

[B26] Friston K. (2005). A theory of cortical responses. *Philos. Trans. R. Soc. Lond. B Biol. Sci.* 360 815–836. 10.1098/rstb.2005.1622 15937014 PMC1569488

[B27] Friston K. (2009). The free-energy principle: a rough guide to the brain? *Trends Cogn. Sci.* 13 293–301. 10.1016/j.tics.2009.04.005 19559644

[B28] Garaizar P. Vadillo M. A. López-de-Ipiña D. Matute H. (2014). Measuring software timing errors in the presentation of visual stimuli in cognitive neuroscience experiments. *PLoS One* 9:e85108. 10.1371/journal.pone.0085108 24409318 PMC3883681

[B29] Gibbon J. (1977). Scalar expectancy theory and Weber’s law in animal timing. *Psychol. Rev.* 84 279–325. 10.1037//0033-295x.84.3.279

[B30] Gibbon J. (1991). Origins of scalar timing. *Learn. Motiv.* 22 3–38. 10.1016/0023-9690(91)90015-Z

[B31] Gibbon J. Church R. M. (1984). “Sources of variance in an information processing theory of timing,” in *Animal Cognition* eds Roitblat H. Bever T. Terrace H. (Hillsdale, MI: Lawrence Erlbaum) 465–488.

[B32] Gilbert C. D. Li W. (2013). Top-down influences on visual processing. *Nat. Rev. Neurosci.* 14 350–363. 10.1038/nrn3476 23595013 PMC3864796

[B33] Grill-Spector K. Henson R. Martin A. (2006). Repetition and the brain: neural models of stimulus-specific effects. *Trends Cogn. Sci.* 10 14–23. 10.1016/j.tics.2005.11.006 16321563

[B34] Hagura N. Kanai R. Orgs G. Haggard P. (2012). Ready steady slow: action preparation slows the subjective passage of time. *Proc. R. Soc. B Biol. Sci.* 279 4399–4406. 10.1098/rspb.2012.1339 22951740 PMC3479796

[B35] Herai T. Mogi K. (2010). Effect of numeric order on subjective duration of following stimulus. *Aust. J. Intell. Inf. Process. Syst.* 11 19–23.

[B36] Horstmann G. Herwig A. (2016). Novelty biases attention and gaze in a surprise trial. *Atten. Percept. Psychophys.* 78 69–77. 10.3758/s13414-015-0995-1 26486643

[B37] Iwasaki M. Tomita K. Noguchi Y. (2017). Non-uniform transformation of subjective time during action preparation. *Cognition* 160 51–61. 10.1016/j.cognition.2016.12.011 28049041

[B38] Kahneman D. (1973). *Attention and Effort.* Englewood Cliffs, NJ: Prentice-Hall.

[B39] Kim E. McAuley J. D. (2013). Effects of pitch distance and likelihood on the perceived duration of deviant auditory events. *Atten. Percept. Psychophys.* 75 1547–1558. 10.3758/s13414-013-0490-5 23801322

[B40] Kok A. de Jong H. L. (1980). The effect of repetition of infrequent familiar and unfamiliar visual patterns on components of the event-related brain potential. *Biol. Psychol.* 10 167–188. 10.1016/0301-0511(80)90013-7 7470516

[B41] Kok P. Rahnev D. Jehee J. F. Lau H. C. de Lange F. P. (2012). Attention reverses the effect of prediction in silencing sensory signals. *Cereb. Cortex* 22 2197–2206. 10.1093/cercor/bhr310 22047964

[B42] Lin Y. J. Shimojo S. (2017). Triple dissociation of duration perception regulating mechanisms: top-down attention is inherent. *PLoS One* 12:e0182639. 10.1371/journal.pone.0182639 28792544 PMC5549740

[B43] Loftus G. R. (1985). Picture perception: effects of luminance on available information and information-extraction rate. *J. Exp. Psychol. Gen.* 114 342–356. 10.1037/0096-3445.114.3.342 3161980

[B44] Loftus G. R. Nelson W. W. Kallman H. J. (1983). Differential acquisition rates for different types of information from pictures. *Q. J. Exp. Psychol.* 35A 187–198. 10.1080/14640748308402124 6681183

[B45] Lupyan G. Clark A. (2015). Words and the world: predictive coding and the language-perception-cognition interface. *Curr. Dir. Psychol. Sci.* 24 279–284. 10.1177/0963721415570732

[B46] Matthews W. J. (2011). Stimulus repetition and the perception of time: the effects of prior exposure on temporal discrimination, judgment, and production. *PLoS One* 6:e19815. 10.1371/journal.pone.0019815 21573020 PMC3090413

[B47] Matthews’ W. J. (2015). Time perception: the surprising effects of surprising stimuli. *J. Exp. Psychol. Gen.* 144 172–197. 10.1037/xge0000041 25494550

[B48] Matthews W. J. Gheorghiu A. I. (2016). Repetition, expectation, and the perception of time. *Curr. Opin. Behav. Sci.* 8 110–116. 10.1016/j.cobeha.2016.02.019

[B49] Matthews W. J. Meck W. H. (2016). Temporal cognition: connecting subjective time to perception, attention, and memory. *Psychol. Bull.* 142 865–907. 10.1037/bul0000045 27196725

[B50] Matthews W. J. Terhune D. B. van Rijn H. Eagleman D. M. Sommer M. A. Meck W. H. (2014). Subjective duration as a signature of coding efficiency: emerging links among stimulus repetition, predictive coding, and cortical GABA levels. *Timing Time Percept. Rev.* 1 1–4. 10.1163/24054496-00101005

[B51] Morrone M. C. Ross J. Burr D. (2005). Saccadic eye movements cause compression of time as well as space. *Nat. Neurosci.* 8 950–954. 10.1038/nn1488 15965472

[B52] New J. J. Scholl B. J. (2009). Subjective time dilation: spatially local, object-based, or a global visual experience? *J. Vis.* 9:4. 10.1167/9.2.4 19271914

[B53] Parducci A. Haugen R. (1967). The frequency principle for comparative judgments. *Percept. Psychophys.* 2 81–82. 10.3758/bf03212467

[B54] Pariyadath V. Eagleman D. M. (2007). The effect of predictability on subjective duration. *PLoS One* 2:e1264. 10.1371/journal.pone.0001264 18043760 PMC2082074

[B55] Pariyadath V. Eagleman D. M. (2012). Subjective duration distortions mirror neural repetition suppression. *PLoS One* 7:e49362. 10.1371/journal.pone.0049362 23251340 PMC3521010

[B56] Peirce J. W. (2007). PsychoPy—psychophysics software in python. *J. Neurosci. Methods* 162 8–13. 10.1016/j.jneumeth.2006.11.017 17254636 PMC2018741

[B57] Poulton E. C. (1968). The new psychophysics: six models for magnitude estimation. *Psychol. Bull.* 69 1–19. 10.1037/h0025267

[B58] Quinn G. P. Keough M. J. (2002). *Experimental Design and Data Analysis for Biologists.* Cambridge: Cambridge University Press 10.1017/cbo9780511806384

[B59] R Development Core Team (2008). *R: A Language and Environment for Statistical Computing.* Vienna: R Foundation for Statistical Computing.

[B60] Rammsayer T. H. (2010). Differences in duration discrimination of filled and empty auditory intervals as a function of base duration. *Atten. Percept. Psychophys.* 72 1591–1600. 10.3758/app.72.6.1591 20675803

[B61] Reggev N. Bein O. Maril A. (2016). Distinct neural suppression and encoding effects for conceptual novelty and familiarity. *J. Cogn. Neurosci.* 28 1455–1470. 10.1162/jocn_a_00994 27315266

[B62] Rose D. Summers J. (1995). Duration illusions in a train of visual stimuli. *Perception* 24 1177–1187. 10.1068/p241177 8577576

[B63] Schindel R. Rowlands J. Arnold D. H. (2011). The oddball effect: perceived duration and predictive coding. *J. Vis.* 11:17. 10.1167/11.2.17 21350128

[B64] Schomaker J. (2015). *What’s New? The Interaction Between Novelty and Cognition.* Amsterdam: Vrije Universiteit.

[B65] Schweitzer R. Trapp S. Bar M. (2017). Associated information increases subjective perception of duration. *Perception* 46 1000–1007. 10.1177/0301006616689579 28084904

[B66] Segaert K. Weber K. de Lange F. P. Petersson K. M. Hagoort P. (2013). The suppression of repetition enhancement: a review of fMRI studies. *Neuropsychologia* 51 59–66. 10.1016/j.neuropsychologia.2012.11.006 23159344

[B67] Seifried T. Ulrich R. (2011). Exogenous visual attention prolongs perceived duration. *Atten. Percept. Psychophys.* 73 68–85. 10.3758/s13414-010-0005-6 21258910

[B68] Sekuler R. Erlebacher A. (1971). The invalidity of “invalid results from the method of constant stimuli”: a common artifact in the methods of psychophysics. *Percept. Psychophys.* 9 309–311. 10.3758/bf03212655

[B69] Shaffer W. O. Shiffrin R. M. (1972). Rehearsal and storage of visual information. *J. Exp. Psychol.* 92 292–295. 10.1037/h0032076 5058950

[B70] Skylark W. Gheorghiu A. (2017). Further evidence that the effects of repetition on subjective time depend on repetition probability. *Front. Psychol.* 8:1915. 10.3389/fpsyg.2017.01915 29163292 PMC5672414

[B71] Stokes M. Thompson R. Nobre A. C. Duncan J. (2009). Shape-specific preparatory activity mediates attention to targets in human visual cortex. *Proc. Natl. Acad. Sci. U.S.A.* 106 19569–19574. 10.1073/pnas.0905306106 19887644 PMC2772815

[B72] Summerfield C. de Lange F. P. (2014). Expectation in perceptual decision making: neural and computational mechanisms. *Nat. Rev. Neurosci.* 15 745–756. 10.1038/nrn3838 25315388

[B73] Tomassini A. Gori M. Baud-Bovy G. Sandini G. Morrone M. C. (2014). Motor commands induce time compression for tactile stimuli. *Proc. Soc. Behav. Sci.* 126 100–101. 10.1016/j.sbspro.2014.02.327 24990936 PMC4112941

[B74] Treisman M. (1963). Temporal discrimination and the indifference interval: implications for a model of the “internal clock.” *Psychol. Monogr.* 77 1–31. 10.1037/h0093864 5877542

[B75] Tse P. U. Intriligator J. Rivest J. Cavanagh P. (2004). Attention and the subjective expansion of time. *Percept. Psychophys.* 66 1171–1189. 10.3758/BF03196844 15751474

[B76] Ulrich R. Nitschke J. Rammsayer T. (2006). Perceived duration of expected and unexpected stimuli. *Psychol. Res.* 70 77–87. 10.1007/s00426-004-0195-4 15609031

[B77] Utzerath C. St John-Saaltink E. Buitelaar J. de Lange F. P. (2017). Repetition suppression to objects is modulated by stimulus-specific expectations. *Sci. Rep.* 7:8781. 10.1038/s41598-017-09374-z 28821808 PMC5562860

[B78] van Wassenhove V. Buonomano D. V. Shimojo S. Shams L. (2008). Distortions of subjective time perception within and across senses. *PLoS One* 3:e1437. 10.1371/journal.pone.0001437 18197248 PMC2174530

[B79] Vangkilde S. Coull J. T. Bundesen C. (2012). Great expectations: temporal expectation modulates perceptual processing speed. *J. Exp. Psychol. Hum. Percept. Perform.* 38 1183–1191. 10.1037/a0026343 22250866

[B80] Wearden J. H. Lejeune H. (2008). Scalar properties in human timing: conformity and violations. *Q. J. Exp. Psychol.* 61 569–587. 10.1080/17470210701282576 18938276

[B81] Yokosaka T. Kuroki S. Nishida S. Watanabe J. (2015). Apparent time interval of visual stimuli is compressed during fast hand movement. *PLoS One* 10:e0124901. 10.1371/journal.pone.0124901 25853892 PMC4390366

